# Co-regulated expression of *HAND2 *and *DEIN *by a bidirectional promoter with asymmetrical activity in neuroblastoma

**DOI:** 10.1186/1471-2199-10-28

**Published:** 2009-04-06

**Authors:** Harald Voth, André Oberthuer, Thorsten Simon, Yvonne Kahlert, Frank Berthold, Matthias Fischer

**Affiliations:** 1 Department of Pediatric Oncology and Hematology and Center of Molecular Medicine Cologne (CMMC), University Children's Hospital, Cologne, Germany; 2 Department of Dermatology, University of Bonn, Bonn, Germany

## Abstract

**Background:**

*HAND2*, a key regulator for the development of the sympathetic nervous system, is located on chromosome 4q33 in a head-to-head orientation with *DEIN*, a recently identified novel gene with stage specific expression in primary neuroblastoma (NB). Both genes are expressed in primary NB as well as most NB cell lines and are separated by a genomic sequence of 228 bp. The similar expression profile of both genes suggests a common transcriptional regulation mediated by a bidirectional promoter.

**Results:**

Northern Blot analysis of *DEIN *and *HAND2 *in 20 primary NBs indicated concurrent expression levels of the two genes, which was confirmed by microarray analysis of 236 primary NBs (Pearson's correlation coefficient r = 0.65). While *DEIN *expression in the latter cohort was associated with stage 4S (p = 0.02), *HAND2 *expression was not associated with tumor stage. In contrast, both *HAND2 *and *DEIN *transcript levels were highly associated with age at diagnosis <12 months (p = 0.001). The intergenic region shows substantial homology in different species (89%, 72% and 53% identity between human and mouse, chicken and zebrafish, respectively) and contains many highly conserved putative transcription factor binding sites. Using luciferase reporter gene constructs, asymmetrical bidirectional promoter activity was found in four NB cell lines: In *DEIN *orientation, an average 3.4 fold increase in activity was observed as compared to the promoterless vector, whereas an average 15.4 fold activation was detected in *HAND2 *orientation. The presence of two highly conserved putative regulatory elements, one of which was shown to enhance *HAND2 *expression in branchial arches previously, displayed weak repressor activity for both genes.

**Conclusion:**

*HAND2 *and *DEIN *represent a gene pair that is tightly linked by a bidirectional promoter in an evolutionary highly conserved manner. Expression of both genes in NB is co-regulated by asymmetrical activity of this promoter and modulated by the activity of two cis-regulatory elements acting as weak repressors. The concurrent quantitative and tissue specific expression of *HAND2 *and *DEIN *suggests a functional link between both genes.

## Background

*HAND2 *is a basic helix-loop-helix transcription factor that is expressed in the developing heart, neural crest derivates and autonomic nervous system including sympathetic neurons, adrenal chromaffin cells and enteric neurons [1]. *HAND2 *plays a key role in the development and differentiation of the sympathetic nervous system by promoting differentiation of neural crest derived precursors into catecholaminergic neurons and maintaining the neural precursor pool of cells [2,3]. Functional analyses showed that *HAND2 *belongs to the group of BMP induced transcription factors that acts downstream of *PHOX2B *and upstream of *GATA2, GATA3, PHOX2A *and the noradrenergic marker genes *Tyrosine Hydroxylase (TH) *and *Dopamine Beta Hydroxylase (DBH) *[3,4]. In addition, *HAND2 *is highly expressed in neuroblastoma (NB), a pediatric tumor arising from undifferentiated migrating sympathetic precursor cells that shows conspicuously divergent biological phenotypes ranging from spontaneous regression to rapid progression and metastasis [5].

Recently, we have reported on the identification of *DEIN*, a novel gene with high expression in NB of children <1 year of age and stage 4S NB, both of which are associated with a good prognosis and spontaneous regression [6]. Given the lack of a putative coding region, *DEIN *was predicted to represent a noncoding RNA whose function in NB and sympathetic nervous system development is yet unclear. *DEIN *is located on chromosome 4q33–34 in a head-to-head orientation with *HAND2 *with both genes being separated by a nucleotide sequence of 228 bp. The close vicinity and divergent configuration of both genes together with their concurrent tissue specific expression pattern suggests a common transcriptional regulation that is mediated by the intergenic sequence. Bidirectional gene organisation was shown to represent a common feature in the human genome with approximately 10% of all genes showing this configuration. Many of these gene pairs were shown to be co-regulated and/or functionally related [7].

This study aimed to examine the potential of the intergenic region to direct transcription in *HAND2 *and *DEIN *orientation, thereby acting as a bidirectional promoter. In addition, conservation and structural features such as putative transcription factor binding sites (TFBS) of the genomic region were analyzed. To test whether the expression of both genes is co-regulated in NB *in vivo *and associated with prognostic factors, expression levels of *DEIN *and *HAND2 *were examined in a cohort of 236 primary NBs of different stages by microarray analysis.

## Results

### Determination of transcriptional start sites and the nucleotide structures of *HAND2 *and *DEIN *transcripts in NB

The human *HAND2 *gene was initially cloned from a human fetal cardiac cDNA library by Russel et al. [8]. The 1.3 kb long consensus sequence derived from cDNA clones AF087940 and AF087941 was shorter than the 2.3 kb transcript detected in Northern Blot, and the authors expected the unaccounted sequence to be located in the 3'-UTR of the gene. However, numerous transcribed sequences containing the coding region of *HAND2 *and extending several 100 bp in 5'-direction have been submitted to GenBank in recent years suggesting that the unaccounted sequence is most probably located upstream of the coding region. In order to determine the transcriptional start site and the full length sequence of the main *HAND2 *transcript in NB, we performed a combined RACE- and RT-PCR approach. The resulting transcript is 2351 bp long and comprises two exons of 1495 and 856 bp (GenBank accession FJ226608). The 5'-sequence of the first exon extends into a CpG island with an average GC-content of 70% which might have impeded the full-length cloning of the human transcript in previous attempts. Our results are well in line with the reported length of 2.3 kb by Northern Blot hybridization from heart tissue [8] and our Northern Blot analyses. The genomic structure of *HAND2 *with a long first and a shorter second exon with the coding region being interrupted by an intron is highly conserved in other species such as mouse, rat, chicken and zebrafish (data not shown). Furthermore, the transcriptional start site of *HAND2 *is highly conserved in all of these species. The 5'-end of FJ226608 is identical with the start sites of mRNA AK122739 and EST DA609347, both of which were derived from NB cell line IMR-32. In contrast, the *HAND2 *reference sequence NM_021973, which has not been experimentally confirmed, lacks two nucleotides at its 5'-end as compared to our transcript. The cloning procedures and the nucleotide structures of the *DEIN *transcript variants have been described in detail previously [6].

### Quantitative expression of *HAND2 *and *DEIN *in primary NB

**Figure 1:**
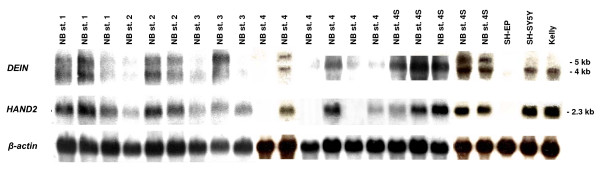
**Northern Blot analysis of *HAND2 *and *DEIN *expression in primary NB and NB cell lines**. Ten μg of total RNA from 20 primary NBs (stage 1, n = 3; stage 2, n = 3; stage 3, n = 3; stage 4S, n = 5; stage 4, n = 6) and NB cell lines SH-EP, SH-SY5Y and Kelly were blotted and hybridized with *HAND2- *and *DEIN*-specific cDNA-probes; β-actin was used as loading control.

To determine whether expression of *HAND2 *and *DEIN *is co-regulated in neuroblastoma, we performed Northern Blot analysis of 20 primary NB with *HAND2*- and *DEIN*-specific cDNA probes. We detected strong expression of both genes in all stage 4S tumors, moderate to low expression in localized tumors, and little or no expression in stage 4 tumors (fig. 1). In NB cell lines, expression of *DEIN *and *HAND2 *was detected in cell lines SH-SY5Y and Kelly, but it was absent in cell line SH-EP. The concurrent transcript levels of *HAND2 *and *DEIN *in primary NB and NB cell lines indicated a co-regulated expression of both genes in NB.

**Figure 2:**
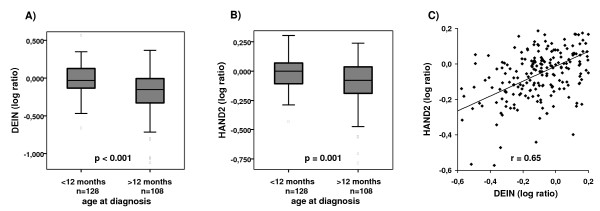
***DEIN *and *HAND2 *expression in 236 primary neuroblastomas as determined by microarray analysis**. Relative expression levels of *DEIN ***(A) **and *HAND2 ***(B) **in neuroblastoma subsets according to age at diagnosis. Scatter Plot showing correlation of relative expression values for *HAND2 *and *DEIN ***(C)**; r, Pearson's correlation coefficient.

To validate these results, expression of *DEIN *and *HAND2 *was analyzed in a set of 236 primary NB samples by microarray analysis (see Additional file 1). Patients diagnosed before the age of one year had significantly higher *DEIN *and *HAND2 *expression values as compared to patients diagnosed later (both p = 0.001; fig. 2A and 2B). Expression of both genes was also significantly higher in children diagnosed before the age of 18 months as compared to older patients (both p = 0.01; data not shown). As expected, *DEIN *expression was significantly higher in tumors of stage 4S than in those of stage 4 (p = 0.02). These results are in line with our previous study that reported on a strong association between *DEIN *expression and stage 4S as well as age < 1 year in a cohort of 121 primary NBs by real-time RT-PCR [6]. However, in contrast to the results of our previous study [6], *DEIN *expression was neither significantly different between stage 4 and localized stages, nor between stage 4S and localized stages in the micoarray analyses (data not shown). This discrepancy might be explained by the high percentage of patients <12 months of age among localized tumors in the microarray cohort (81/119 patients, 68%). *HAND2 *mRNA levels were not associated with either stage, which is in agreement with a study of Gestbloom et al., who reported that *HAND2 *is expressed independently of tumor stage or differentiation in NB [5]. Nevertheless, *HAND2 *and *DEIN *expression values were well correlated in our study (Pearson's correlation coefficient r = 0.65, fig. 2C). Together, these findings support the hypothesis that expression of *HAND2 *and *DEIN *is co-regulated in primary NB.

### Characterization of the putative bidirectional promoter sequence between *HAND2 *and *DEIN*

**Figure 3:**
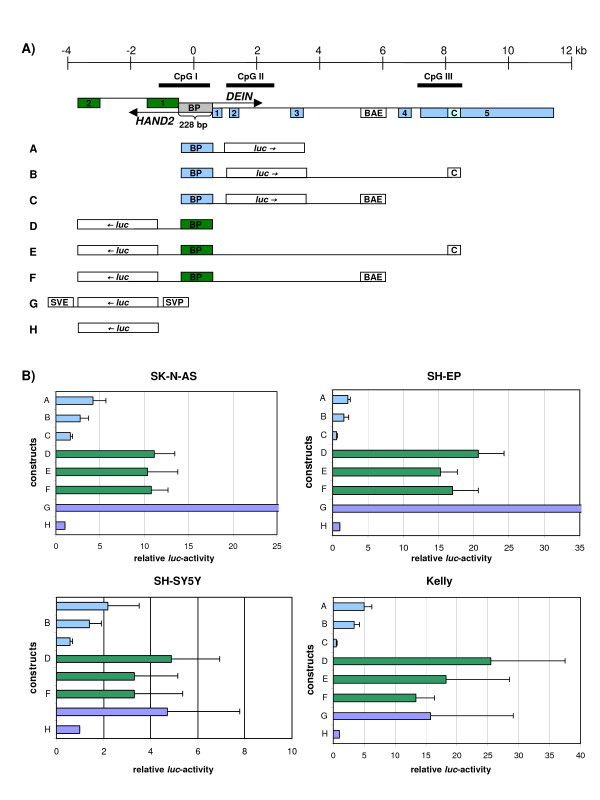
**Genomic organization of the human *HAND2*-*DEIN *locus on 4q33–34 and luciferase reporter-gene constructs A-H (A); Relative luciferase activity of constructs A-H in NB cell lines SK-N-AS, SH-EP, SH-SY5Y and Kelly (B)**. Boxes and lines indicate exons and introns, respectively. Arrows indicate direction of transcription. Blue bars, *DEIN *orientation; green bars, *HAND2 *orientation; BP, Bidirectional Promoter; *luc*, *luciferase*; BAE, *HAND2 *Branchial Arch Enhancer; C, putative cis-regulatory element; SVE/SVP, SV40 enhancer- and promoter-elements, respectively. Values of transfection experiments are shown as the mean ± standard deviation of 3 independent experiments. Values are normalized to the activity of the pGL3-Basic vector set as 1.

The putative promoter between *HAND2 *and *DEIN *is located within a CpG island (GC-content of 70%) that extends into the first exon of *HAND2 *(fig. 3). The intergenic region shows high evolutionary conservation with 89, 71 and 53% nucleotide identity of the human sequence in comparison to mouse, chicken and zebrafish, respectively. The high degree of homology is comparable to that of the coding region of *HAND2 *which shows 92, 88 and 68% identity of the human sequence in comparison to mouse, chicken and zebrafish, respectively.

**Figure 4:**
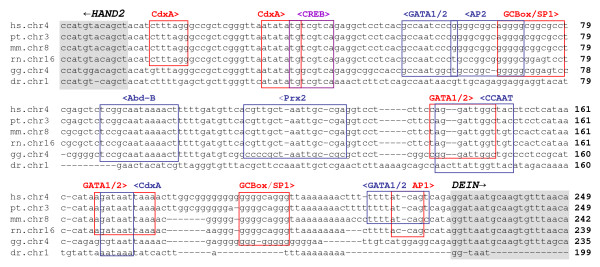
**Sequence alignment of the complete nucleotide sequence of the bidirectional promoter between *HAND2 *and *DEIN *in human (hs), chimpanzee (pt), mouse (mm), rat (rn), chicken (gg) and zebrafish (dr)**. First exons of *HAND2 *and *DEIN *are shaded in grey. Direction of transcription is indicated by black arrows. Nucleotides are numbered relative to the first nucleotide of the bidirectional promoter. Putative Trancription Factor Binding Sites (TFBS) are boxed. The directionality of TFBS is indicated by arrowheads and color of boxes (<, blue, *HAND2*-orientation; >, red, *DEIN*-orientation). SP-1, specificity protein 1; AP-1 and AP-2, activator protein 1 and 2; CdxA, CdxA homeobox gene; Abd-B, Abdominal-B; CREB, cAMP response element-binding protein; Prx2, paired related homeobox 2.

No TATA-Box, but various other highly conserved potential transcription regulatory elements were found in the bidirectional promoter (fig. 4). These include two GC-boxes with SP-1 sites and one AP-1 site in *DEIN*-orientation as well as one AP-2 site in *HAND2*-orientation. Four binding sites for zinc finger transcription factors GATA-1 or -2 (two each in *DEIN *and *HAND2 *orientation, respectively) were detected. We also found four binding sites for homeobox transcription factors including three binding sites for CdxA (one in *HAND2 *orientation and two in *DEIN *orientation), and one binding site each for Abd-B and Prx2 in *HAND2 *orientation. Finally, a highly conserved site for cAMP response element-binding protein (CREB) in both orientations and a highly conserved centrally located CCAAT box in *HAND2 *orientation were detected. Evolutionary conservation was found for the CDxA, CREB and the CCAAT binding sites in all vertebrate species investigated, while SP1, AP1 and 2, Abd-B and GATA binding sites were only conserved in mouse and/or chicken.

### Asymmetrical activity of the bidirectional promoter between *DEIN *and *HAND2*

To test the ability of the putative bidirectional promoter to activate transcription in *DEIN *and *HAND2 *orientation, it was cloned separately or together with two other highly conserved putative regulatory elements in both orientations into a *luciferase *reporter vector. A total of six reporter gene constructs were transfected into four neuroblastoma cell lines (summarized in fig. 3). It was demonstrated that the bidirectional promoter is able to drive expression in both *HAND2 *and *DEIN *direction in all cell lines with ~4 fold higher activity in *HAND2 *orientation than in *DEIN *orientation. In *DEIN *orientation the promoter caused a 2.2 – 5 fold activation (mean value, 3.4) as compared to the promoterless vector (p < 0.01 for all cell lines except SH-SY5Y, which is probably due to the lower transfection efficiency of SH-SY5Y). A 4.9 – 25.5 fold increase in activity (mean value, 15.4) was detected for the promoter in *HAND2 *orientation in all cell lines as compared to the promoterless vector (p < 0.01 for all cell lines).

To test whether promoter activity is influenced by either of two other highly conserved sequence fragments that are located within the genomic sequence covered by *DEIN*, we cloned these elements either upstream or downstream of the *luciferase *gene in the respective orientations relative to the bidirectional promoter according to their actual genomic position (constructs B, C, E and F, fig. 3A). The presence of a 90 bp long regulatory element that was shown to act as enhancer for *HAND2 *expression in branchial arches [9] decreased *luciferase *activity in both *DEIN *and *HAND2 *orientations. For construct C, an average 2.6 fold reduction in activity was detected as compared to construct A (p = 0.05 for SK-N-AS, SH-EP and Kelly; not significant [n.s.] for SH-SY5Y). For construct F, an average 4.4 fold reduction in activity was detected as compared to construct D (p = 0.05 for Kelly; n.s. for SH-SY5Y, SH-EP and SK-N-AS).

Another highly conserved sequence element of 302 bp located in exon 5 of *DEIN*, which has been suggested to represent a cis-regulatory element for *DEIN *and/or *HAND2 *expression [6], also exerted weak repressor activity on both promoters. For construct B, we detected an average 1.1 fold reduction in activity as compared to construct A (p < 0.05 for SH-EP, SK-N-AS and Kelly; n.s. for SH-SY5Y). For construct E, we detected an average 3.2 fold reduction as compared to construct D (p < 0.05 for SH-EP; n.s. for SH-SY5Y, SK-N-AS and Kelly).

## Discussion

In this study, we report on the molecular characterization of a highly conserved intergenic nucleotide sequence between *HAND2 *and *DEIN *which functions as a bidirectional promoter. Bidirectional gene configuration, defined as two divergently transcribed genes that are separated by less than 1 kb, is a common architectural feature in the human genome and was shown to account for co-regulated and/or tissue specific expression of functionally related genes [10]. Most of these genes have been reported to belong to the group of housekeeping genes many of which are involved in DNA repair and replication [7]. In recent years, other non-homologous bidirectional gene pairs were identified with functions in senescence [11], antigen presentation [12], brain disease [13,14], oncogenesis or tumor supression [15-17].

### Structure and conservation of the bidirectional promoter

The majority of bidirectional promoters are less than 300 bp in length, have a median GC-content of 66% and associate with CpG-islands in 75% [7]. All these features are present in the common promoter region of *HAND2 *and *DEIN*, which is 228 bp long, contains 13 CpG sites and has a GC-Content of 70%. Interestingly, the distance between *HAND2 *and *DEIN *remained stable throughout evolution with only 14 and 7 additional nucleotides in the human sequence as compared to chicken and mouse, respectively. Although there is no systematic evaluation on the alteration of the genomic distance between bidirectional gene pairs throughout evolution, we found examples reporting a far more dramatic increase of several hundreds of bp [11]. The constancy of the intergenic distance between *HAND2 *and *DEIN *might thus be indicative of the selective pressure that was acting to maintain the close distance of the genes over a long evolutionary period.

The nucleotide sequence, exon number and transcript structure including intron-exon boundaries of *HAND2 *are highly conserved among different species including chicken and zebrafish. In contrast, *DEIN *is only conserved in mouse and chicken but not zebrafish, and shows an overall lower conservation that decreases in 3'-direction with the highest conservation in exons 1 and 2 and a region in exon 5 [6]. Numerous transcribed sequences matching nearly all exons of *DEIN *were found in mouse and chicken, but not in zebrafish. Tissue sources for these sequences included typical *HAND2 *expressing tissues such as heart, and embryonic or fetal tissues such as trophoblast and developing sympathetic ganglia, respectively [6]. We conclude from these findings that (1) *DEIN *has probably first evolved after zebrafish in higher vertebrates, and that (2) bidirectional transcription of *HAND2 *and *DEIN *is probably evolutionary conserved in higher vertebrates such as rodents and birds. The latter conclusion is further supported by large-scale comparative analyses of bidirectional promoters in vertebrates using orthology assignments, according to which many bidirectional promoters have emerged after the divergence of birds and fish [18].

### Asymmetrical activity of the bidirectional promoter in NB cell lines

The activity of the bidirectional promoter in four NB cell lines was shown to be asymmetric with strong activity in *HAND2 *and moderate activity in *DEIN *orientation. Asymmetric activity has been reported previously for other divergently transcribed gene pairs [13,19].

The comparatively low activity of the promoter in *DEIN *orientation is surprising regarding the strong expression of *DEIN *in primary NB as indicated by SAGE, Northern Blot and RT-PCR analysis (fig. 1 and [6]). *DEIN *expression might possibly require additional cis-regulatory elements that were not included in our constructs. Alternatively, activation of the promoter in *DEIN *orientation could also depend on transcriptional activators absent in NB cell lines, which are derived from rapidly progressing tumors characterized by rather low *DEIN *mRNA levels [6]. Transcript levels of both genes might also be further modified by post-transcriptional mechanisms such as mRNA modification or degradation.

Two other highly conserved sequence elements, one of which was shown to function as an enhancer for *HAND2 *transcription in branchial arches [9], appear to act as weak repressors for both *DEIN *and *HAND2 *transcription in NB. However, the *in vitro *repressive activity could also be a result of steric inhibition of transcription factors, since the genetic distance between the two regulatory elements and the promoter in the genome is about 6 – 8 kb, whereas it is only 44 bp – 1.9 kb in the luciferase reporter constructs.

### Transcriptional regulation and putative functional link of *HAND2 *and *DEIN *in NB

Tissue specific regulation of *HAND2 *expression by cis-regulatory elements located in its upstream genomic region has been extensively studied in heart and branchial arches, but to date, genomic elements that are critical for *HAND2 *expression in the developing sympathetic nervous system have not been identified. In heart, *HAND2 *transcription depends on an enhancer element consisting of two conserved *GATA *sites that are dispersed over a genomic region of 1.5 kb between exon 3 and 4 of *DEIN *with an overall low conservation [20]. In branchial arches, *HAND2 *expression depends on endothelin-signaling via *Dlx6 *which binds to a highly conserved 200 bp long enhancer element located between exon 3 and 4 of *DEIN *[9]. According to McFadden et al. who studied regulatory elements for *HAND2 *expression in mouse, the genomic region 11 kb upstream of *hand2 *did not direct expression in sympathetic ganglia [20]. This result seems to be in contrast with our finding of a strong transcriptional activation of a reporter gene by the 228 bp upstream region of *HAND2 *in human NB cells that are suggested to derive from sympathicoadrenal precursors.

**Figure 5:**
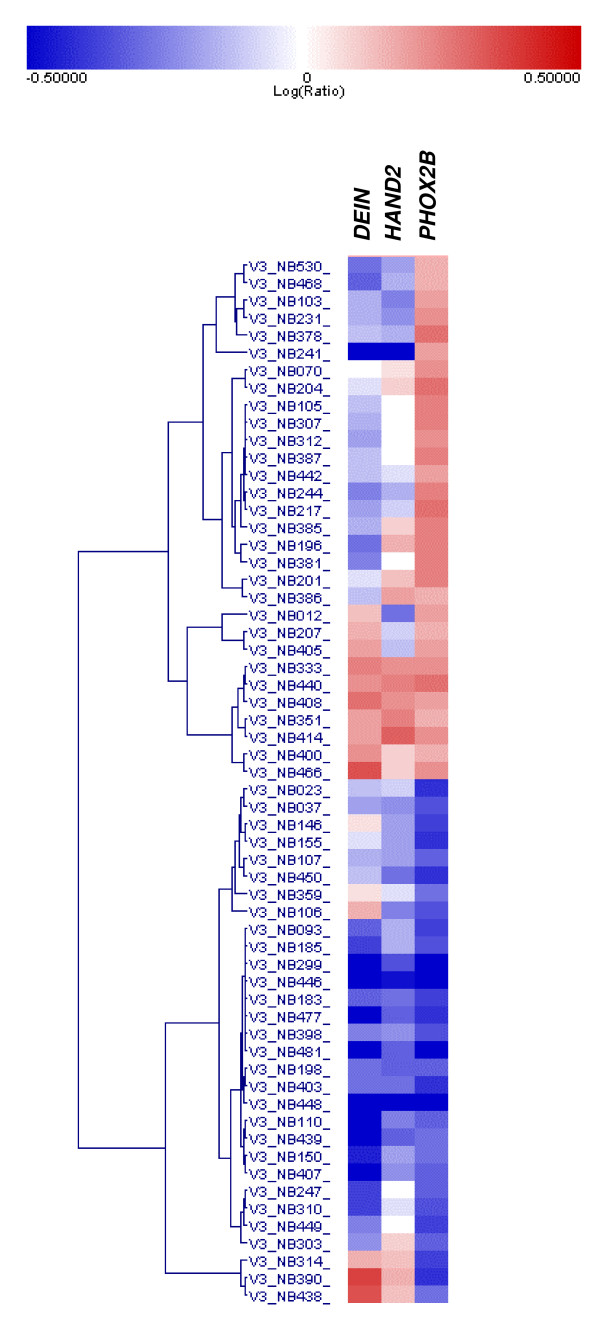
**Hierarchical cluster analysis of 60 primary neuroblastomas with high and low *PHOX2B *expression levels according to expression of *DEIN, HAND2 *and *PHOX2B *as determined by microarray analysis**. Lines represent patients, columns represent genes. Gene expression levels are visualized as log-values ranging from blue (-0.5) to red (+0.5).

Our data indicate that the bidirectional promoter is an important element for *HAND2 *expression in sympathicoadrenal tissues, although the exact transcriptional activators have not been specified yet. It has been demonstrated earlier that *Phox2b *knockout mice lack *Hand2 *expression [21]. These data strongly suggest that *PHOX2B*, a master gene required for the development and differentiation of autonomic neurons [22], is an upstream activator of *HAND2 *in the developing sympathetic nervous system [2,3,23]. Expression analyses of *PHOX2B, HAND2 *and *DEIN *in NB cells lines SH-SY5Y and SH-EP, two well-studied subclones of SK-N-SH with a neuronal-like and epithelial-like phenotype, respectively [24], further suggest that not only *HAND2*, but also *DEIN *is a target gene of *PHOX2B*: In cell line SH-EP, *PHOX2B *expression was shown to be absent [25], and likewise, neither *HAND2 *nor *DEIN *was expressed in SH-EP cells according to our Northern Blot analysis (fig. 1); in contrast, all three genes are expressed in cell line SH-SY5Y ([25] and fig. 1). In order to investigate whether *DEIN *and *HAND2 *are possible downstream targets of *PHOX2B *in primary NB, we simultaneously examined *DEIN *and *HAND2 *mRNA levels in sixty selected tumors with high and low *PHOX2B *expression values using microarrays (fig. 5). Tumors of both subgroups showed largely concordant expression levels of *DEIN *and *HAND2*. However, whereas low expression levels of *PHOX2B *were consistently associated with low mRNA levels of *DEIN *and *HAND2 *in the vast majority of cases, the degree of correlation appeared to be lower for tumors with high *PHOX2B *expression levels. These observations provide further evidence that *PHOX2B *may act as an upstream activator of both *DEIN *and *HAND2*, although additional factors may be necessary to induce expression of these genes in NB. Taking the presence of multiple conserved binding sites for homeobox transcription factors within the bidirectional promoter and the concurrent expression of *HAND2 *and *DEIN *in NB into account, it is tempting to speculate that *PHOX2B *could actually represent a direct transcriptional activator for *HAND2 *and *DEIN *via the common promoter sequence.

The known *PHOX2B *downstream targets *HAND2 *and *PHOX2A *were shown to promote differentiation of sympathicoadrenal cells and expression of noradrenergic marker genes. In NB cell lines, forced over-expression of wild-type *PHOX2B *was shown to suppress cell proliferation and promote differentiation [26]. As another hypothetical *PHOX2B *target gene, *DEIN *might be involved in these cellular processes. This is particularly interesting in the light of the strong *DEIN *expression in tumors of stage 4S and those of children diagnosed <1 year of age. Since these tumors are associated with a good prognosis and often follow spontaneous regression, they have been speculated to represent embryonal remnants rather than malignant tumors [27].

Bidirectional gene pairs have been shown to be functionally related, e.g. in DNA repair [7], senescence [11] or brain disease [13,14]. The concurrent quantitative and tissue specific expression of both genes ([6], fig. 1 and 2C) suggests that *HAND2 *and *DEIN *are regulated by common transcriptional mechanisms in a restricted range of tissues during early developmental stages. The bidirectional promoter probably plays an important role in coordinating *HAND2 *and *DEIN *expression in a similar spatial and temporal fashion. However, the functional relevance of the linkage between *DEIN *and *HAND2 *expression by the bidirectional promoter requires further investigation, e.g. functional studies on *DEIN *and experiments elucidating the transcription factors that bind to the promoter region.

## Conclusion

*HAND2 *and *DEIN *represent a gene pair that is tightly linked by a bidirectional promoter in an evolutionary highly conserved manner. The activity of the promoter in NB cell lines is asymmetric with strong activity in *HAND2 *and moderate activity in *DEIN *orientation. Expression studies strongly suggest co-regulation of both genes, and their high expression in tissues corresponding to the developing sympathetic nervous system suggests a functional link between *HAND2 *and *DEIN*. Co-regulated and tissue specific expression are likely both mediated by the bidirectional promoter, which appears to be evolutionary highly conserved in rodents and birds. Concordant expression levels of *HAND2*, *DEIN *and *PHOX2B *in many primary NB and NB cell lines suggest that *PHOX2B *acts as an upstream transcriptional activator for both genes. Regarding the key role of *HAND2 *in the differentiation of neural crest derived precursors into sympathetic neurons and the high expression of *DEIN *in spontaneously regressing NB, *DEIN *could be instrumental in biological phenomena like regression and differentiation, which are characteristic for favourable NB and the developing sympathetic nervous system.

## Methods

### 3'- and 5'-RACE-PCR

To identify the 5'-ends of *HAND2 *and *DEIN*, 5'-RACE-PCR was performed using the SMART™RACE cDNA Amplification Kit (Clontech, Mountain View, USA) with the gene specific primers GSPH2 for *HAND2 *and GSPD1 for *DEIN*. Cloning of the 3'-end of *HAND2 *was performed using the SMART™RACE cDNA Amplification Kit (Clontech) with the gene specific primer GSPH1 (table 1).

**Table 1:** Oligonucleotides used as primers in this study

*method*	*name*	*nucleotide sequence*
3'-RACE	GSPH1	5'-CATGGACCTGCTGGCCAAGGACG-3'

5'-RACE	GSPH2	5'-CTGAATGAGCCTTGGAGCTCGAAG-3'
	GSPD1	5'-GCCAATCTGCAGTTTGTCTTTCTG-3'

reporter gene constructs	BPF	5'-TTTTAAGCTTACATCTTTAGGGCCGCTCG-3'
	BPR	5'-TTTTAAGCTTGTTAAACACTTGCATTATCCTCTG-3'
	CDF	5'-TTTTGGATCCACCTTTCGCCGGAGGCGA-3'
	CDR	5'-TTTTGGATCCAGCGAGCGGCTGCAGATTTG-3'
	CHF	5'-TTTTACGCGTACCTTTCGCCGGAGGCGA-3'
	CHR	5'-TTTTACGCGTAGCGAGCGGCTGCAGATTTG-3'
	BDF	5'-TTTTGGATCCGTTTGTAATAAGAGAATGACCGAA-3'
	BDR	5'-TTTTGGATCCAGGTCTCCTTGGTAATTTGGGTAC-3'
	BHF	5'-TTTTACGCGTGTTTGTAATAAGAGAATGACCGAA-3'
	BHR	5'-TTTTACGCGTAGGTCTCCTTGGTAATTTGGGTAC-3'

RT-PCR & Northern Blot	F1	5'-AGCTGTACATGGAGATCTTGC-3'
	F2	5'-AAAATCAAGACCCTGCGCCTG-3'
	F3	5'-GCGAAATGAGTCTGGTAGGTG-3'
	F4	5'-CGACCCATGTAATATGTAACA-3'
	F5	5'-AATGGGATTCTCTATTTGTGCTG-3'
	F6	5'-GAAGGCACAGATCATTCATGG-3'
	R1	5'-CTCACTGTGCTTTTCAAGATTTC-3'
	R2	5'-CTTGTCGTTGCTGCTCACT-3'
	R3	5'-CACAGTGGTTTATTGAATACTTAC-3'
	R4	5'-CACCT ACCAGACTCA TTTCGC-3'
	R5	5'-TCAGCTAGAAAACTGTATAAGAG-3'
	R6	5'-ACCACAAGCAGTCTCATGGGA-3'
	R7	5'-CAGTAAAAAAAACAGTTTGAAAGGC-3'

Forward primers are denoted F, reverse primers are denoted R; GSPH1-GSPD1 are gene specific primers for 3'- and 5'-RACE. BPF-BHR were used for amplification of fragments for subsequent cloning of reporter gene constructs A–F (restriction sites are underlined). F1–6 and R1–7 are primers used for RT-PCR and generation of Northern Blot probes.

### Synthesis of cDNA, cloning and sequencing of PCR products

Generation of cDNA used for 5'- and 3'-RACE-PCR and RT-PCR was performed with 0.4 – 2 μg of poly(A)-RNA of a primary stage 4S neuroblastoma. For RT-PCR, cDNA-synthesis was performed with the QIAGEN LongRange 2Step RTPCR kit according to the manufacturer's protocol. Cloning of PCR products was carried out using the TOPO TA^®^-Vector (Invitrogen). Sequencing was performed using the BigDye terminator sequencing kit, Version 3.1 (Applied Biosystems).

### Reverse transcriptase (RT)-PCR

PCR was carried out in a total volume of 50 μl containing 1 μl first strand cDNA, 2 U HiFi Platinum-Taq DNA Polymerase (Invitrogen), 125 nM sense and anti-sense primer each, 20 mM Tris-HCl (pH 8.4), 50 mM KCl, 200 nM dNTPs each and 1.5 mM MgSO_4 _(Invitrogen). Cycling conditions consisted of a single denaturation step at 95°C for 3 min, followed by 35 cycles of 95°C for 1 min, 58°C for 1 min, 72°C for 11.5 min and a final extension step at 72°C for 15 min. For the amplification of overlapping subfragments of *HAND2*, the primer combinations F1 + R4 and F3 + R3 (table 1) were used. PCR products were visualized on a 2% agarose gel. As a negative control, RT-PCR reactions were performed containing 1 μl of first strand cDNA reactions without reverse transcriptase to exclude amplification of contaminating genomic DNA.

### Northern Blot analysis

Ten μg of total RNA from 20 randomly selected primary NB specimen (stage 1, n = 3; stage 2, n = 3; stage 3, n = 3; stage 4S, n = 5; stage 4, n = 6) and 3 NB cell lines (SH-EP, SH-SY5Y and Kelly) were size-fractioned on a 1% denaturating formaldehyde agarose gel and transferred onto a nylon membrane (Roche Diagnostics, Mannheim, Germany) using Northern Max One-Hour Transfer Buffer (Ambion, Cambridgeshire, UK). Specific cDNA-probes for *DEIN*, *HAND2 *and *β-actin *were generated by RT-PCR. Primers used for amplification of specific cDNA-probes were F4 + R5 for *DEIN*, and F2 + R3 for *HAND2 *(table 1). Blots were hybridized overnight at 42°C in UltraHyb Hybridization Buffer (Ambion) with high sensitivity strippable DNA probes labelled with [α^32^P]-dATP (Strip-EZ DNA kit, Ambion). After hybridization, membranes were washed, air-dried and exposed to Kodak BioMax MR-1 films (Amersham, Freiburg, Germany).

### Microarray analysis and statistical analysis of expression data

Gene expression profiles from 236 patients (localized stages, n = 119; stage 4S, n = 28; stage 4, n = 89; age <1 year, n = 128; age >1 year, n = 108) were generated as dye-flipped dual-color replicates using customized 11 K microarrays as described [28]. This set of patients did neither overlap with the cohort of the Northern Blot analysis nor with the cohort of our previous study [28]. Variables of interest (stage and age at diagnosis) and relative *DEIN *and *HAND2 *expression levels were compared using Chi-square-test, Kruskal-Wallis test or Mann-Whitney-U-test where appropriate. Correlation of relative expression values was calculated with Pearson's correlation coefficient.

Hierarchical clustering analyses was performed using the Rosetta Resolver Software (Version 7.2; Rosetta Inpharmatics LCC, Seattle). From the whole set of 236 patients, groups with highest (n = 30) and lowest (n = 30) *PHOX2B *expression were picked and used for the clustering. The analysis was performed using Complete Linkage and Cosine Correlation as metric variables.

### Sequence analysis

The GC-content and CpG islands were determined using CpGplot . Homology analysis was performed by comparing sequences of different species with the human sequence of the bidirectional promoter using the ALIGN-webtool, which calculates a global alignment between two sequences . Prediction of putative TFBS was performed using the program Motif .

### Reporter gene constructs and luciferase assays

The intergenic region between *HAND2 *and *DEIN *was amplified by PCR from human genomic DNA with the primers BPF and BPR (table 1) containing a 10 bp cloning adaptor with a *Hin*dIII-restriction site. It was then cloned in both orientations into the *Hin*dIII-site of the pGL3-Luciferase reporter Vector (constructs A and D, fig. 3; Promega, Darmstadt, Germany).

The 302 bp long putative cis-regulatory element "C" was amplified by PCR with the primers CDF and CDR and cloned downstream of the luciferase gene into the BamHI-site of the *DEIN*-promoter vector (construct B, fig. 3). For cloning of construct E, "C" was amplified with the primers CHF and CHR and cloned upstream of the luciferase gene into the MluI-site of the *HAND2*-promoter vector (construct E, fig. 3). The 90 bp long enhancer for *HAND2*-expression in branchial arches (BAE) was PCR amplified with the primers BDF and BDR and cloned downstream of the luciferase gene into the BamHI-site of the *DEIN*-promoter-vector (construct C, fig. 3). For cloning of construct F, "BAE" was amplified with the primers BHF and BHR and cloned upstream of the luciferase gene into the MluI-site of the *HAND2*-promoter vector (construct F, fig. 3).

All constructs were sequenced with the BigDye Terminator Kit (PE Applied Biosystems, Foster City, USA) to ensure the correct orientation and nucleotide sequence. NB cell lines SH-EP, SK-N-AS, SH-SY5Y and Kelly were cultured in RPMI medium supplemented with 10% FCS. Cells were transfected using Lipofectamin 2000 (Invitrogen, Karlsruhe, Germany) following the manufacturer's instructions. Approximately 3 × 10^4 ^cells were transferred into 96-well plates and transfected with a mixture containing 1 μg of the respective reporter construct and 2 ng of pRL-CMV (Promega), which contains the Renilla luciferase gene under the cytomegalovirus SV 40 promoter and enhancer serving as an internal control. Cells were harvested and lysed 27–30 h after transfection and the luciferase activity was detected using the Dual Luciferase Reporter Assay Kit (Promega) and measured with a luminometer (Mitras, Berthold, Germany) according to the manufacturer's instructions. The luciferase activity of the reporter plasmids was normalized to the renilla luciferase acitivity. Each transfection experiment was carried out three times in duplicate and a total of four measurements for each construct were performed. Student's t-test was used to calculate the statistical significance between the relative activities of the various constructs compared to that of the promoterless vector.

## Authors' contributions

HV is responsible for the conception of the study, the performance of RT-PCR, luciferase reporter assays and sequence analyses, and the writing of the manuscript. AO performed the analysis of microarray expression data. TS carried out the statistical analysis of expression data. YK performed Northern Blots and luciferase reporter assays. FB provided clinical data and contributed to critically revise the manuscript. MF coordinated the study, participated in the design and the writing of the manuscript, and revised it critically for important intellectual content. All authors read and approved the final manuscript.

## Supplementary Material

Additional File 1 **The table indicates expression values (log ratios) of *HAND2*, *DEIN *and *PHOX2B *in 236 primary NBs as determined by micoarray analysis, patient ID, stage, *MYCN*-status (1, single copy; A, amplified) and age at diagnosis (days).** Click here for file
